# Cluster headache: understandings of current knowledge and directions for whole process management

**DOI:** 10.3389/fneur.2024.1456517

**Published:** 2024-08-21

**Authors:** Xiao-Hu Xu, Yi-Ming Li, Li-Na Ren, Xiao-Fan Xu, Yi-Long Dai, Cheng-Qiang Jin, Rui-Rui Yang

**Affiliations:** ^1^Department of Clinical Medicine, Jining Medical University, Jining, Shandong, China; ^2^Medical Laboratory, Affiliated Hospital of Jining Medical University, Jining Medical University, Jining, Shandong, China; ^3^Neurology Department, Shandong Provincial Hospital Affiliated to Shandong First Medical University, Jinan, China

**Keywords:** cluster headache, pathogenesis, epidemiology, manifestation, whole process management

## Abstract

Cluster headache (CH) is a common primary headache that severely impacts patients’ quality of life, characterized by recurrent, severe, unilateral headaches often centered around the eyes, temples, or forehead. Distinguishing CH from other headache disorders is challenging, and its pathogenesis remains unclear. Notably, patients with CH often experience high levels of depression and suicidal tendencies, necessitating increased clinical attention. This comprehensive assessment combines various reports and the latest scientific literature to evaluate the current state of CH research. It covers epidemiology, population characteristics, predisposing factors, and treatment strategies. Additionally, we provide strategic insights into the holistic management of CH, which involves continuous, individualized care throughout the prevention, treatment, and rehabilitation stages. Recent advances in the field have revealed new insights into the pathophysiology of CH. While these findings are still evolving, they offer a more detailed understanding of the neurobiological mechanisms underlying this disorder. This growing body of knowledge, alongside ongoing research efforts, promises to lead to the development of more targeted and effective treatments in the future.

## Introduction

**Figure 1:**
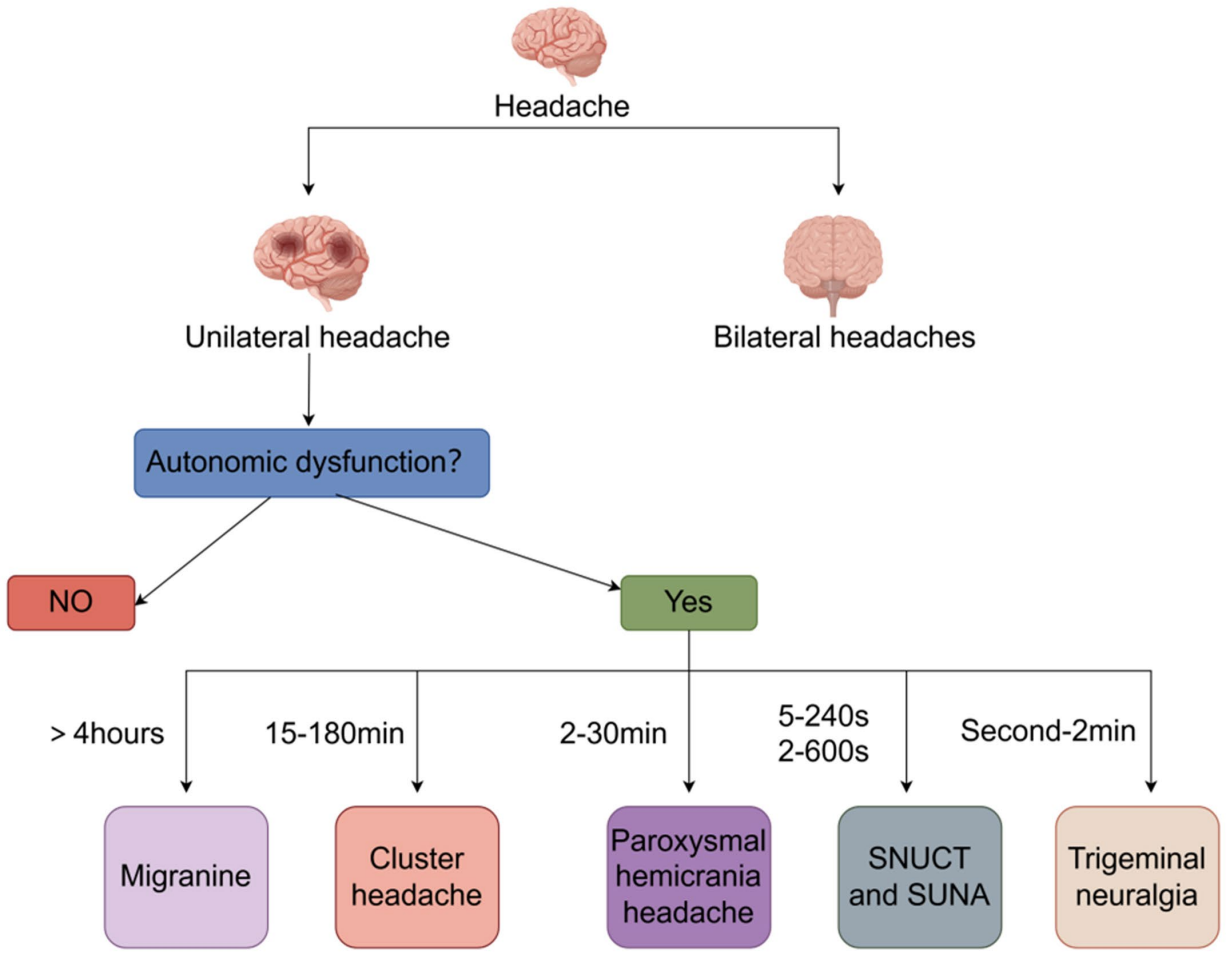
Identification of cluster headache by location, nature, and duration of pain.

Headaches are one of the most common symptoms of human suffering. However, if the headaches recur more frequently than expected and there are no triggers, trauma, or underlying disease, they are categorized as a disorder. Of these disorders, cluster headaches (CH) are one of the most severe forms (1). In the typical form of the disorder, severe unilateral headaches that occur several times a day may be a basis for differentiating CH from migraine (2, 3). The Headaches usually occur in the supraorbital, retroorbital, and temporal regions and are caused by deep cranial, and the frequency of attacks can start out as occurring every other day and then increase to a maximum of eight per day, with attacks having a diurnal rhythm, favoring nocturnal attacks (4). In addition to headache, patients with cluster headache may present with autonomic symptoms such as tearing, conjunctival injection, nasal leakage, pupil constriction, ptosis, hyperhidrosis, eyelid edema and flushing (5). Alarmingly, cluster headache is a highly disabling headache disorder in which patients typically exhibit depressive symptoms, are more prone to suicidal thoughts and behaviors than the general population and have a significantly reduced quality of life (6). Therefore, we review the pathogenesis, diagnosis, and treatment of cluster headache and emphasize the management of cluster headache throughout the course of the disease in an attempt to improve the understanding of cluster headache and the quality of life of patients (Figure 1).

## Epidemiology

### Incidence and prevalence

Cluster headaches are influenced by several variables, including age, gender, and geographic location. One in 1,000 people suffers from cluster headaches, with a prevalence of 0.1%–0.2%, according to epidemiologic surveys (7). CH is the most commonly diagnosed rare headache, and in a study involving 20,083 people attending a headache center, 461 cases were diagnosed with rare headache, of which 234 (1.2%) were diagnosed with episodic cluster headache, and 39 (0.2%) patients were newly diagnosed with cluster headache, which accounted for 59.2% of the rare headaches (8). Not only is CH the most commonly diagnosed rare headache, it also has a serious impact on patients’ quality of life, and the disorder is highly misdiagnosed, with actual incidence and prevalence likely to be higher.

In addition, there are variations in incidence and prevalence in different regions. For example, some studies have found that the prevalence of cluster headaches is higher in the Nordic region and lower in Asia (9). This difference may be related to factors such as geography, lifestyle, and climate.

### Affecting factors

#### Age

Cluster headaches are most common in specific age groups, especially young and middle-aged people between the ages of 20 and 40. There is a significantly higher prevalence of cluster headache in men in this age group compared to other age groups (10). The incidence of cluster headache decreases with age, which may be related to age-related physiologic changes and lifestyle changes.

#### Gender

**Figure 2:**
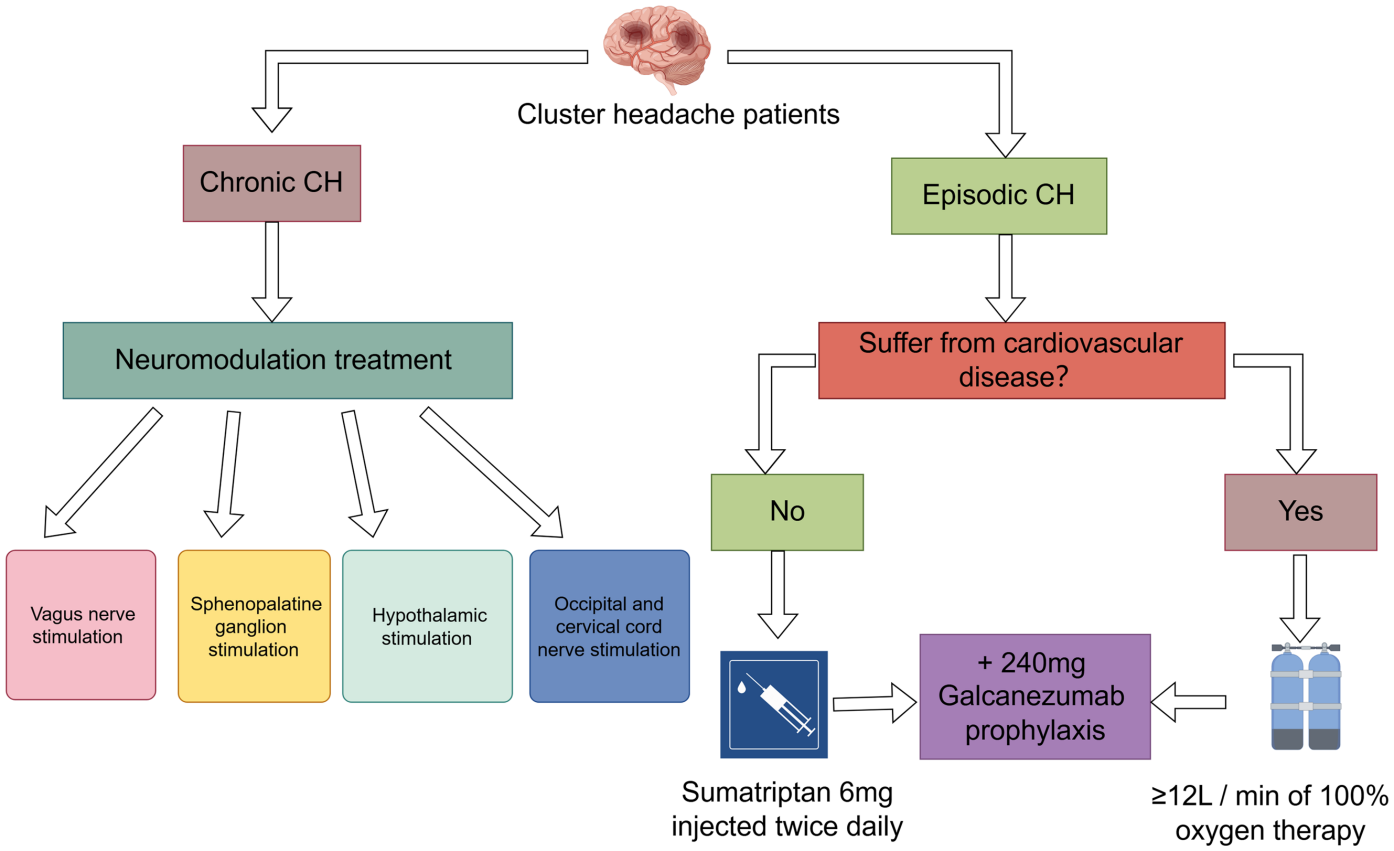
Treatment of cluster headache.

The proportion of men among cluster headache sufferers is significantly higher than that of women; 20 years ago, the ratio of men to women was about 4.7:1, and although the ratio of men to women has declined, there are still far more men than women. This may be related to the differences between men and women in terms of social roles, work pressures, and psychological endurance (11). Women may have a lower incidence due to estrogen (12). Therefore, men should be more aware of the prevention and treatment of cluster headaches than women (Figure 2).

#### Genetic factors

Studies have shown that cluster headache has a significant family aggregation. Patients with a family history of cluster headaches have a higher incidence, and it is more common to have multiple generations of patients in the family. A first-degree relative with cluster headaches increased the risk to the patient by 5 to 18 times, while a second-degree relative with cluster headaches increased the risk by 1 to 3 times (13, 14). And it has been shown that symptoms such as nasal congestion and conjunctival congestion are more prevalent in familial cluster headaches than in non-familial cluster headaches (15). In addition, a genetic study of CH found that a total of seven protein-coding genes (DUSP10, MERTK, FTCDNL1, FHL5, WNT2, PLCE1, and LRP1) were associated with the development of cluster headache (16). This suggests that genetic factors play a significant role in the development of cluster headache. Further research will help to reveal the specific genes and genetic mechanisms associated with cluster headache, thus providing innovative ideas and methods for the prevention and treatment of the disease.

#### Lifestyle habits and environmental factors

A wide range of lifestyle habits are thought to be associated with an increased risk of developing cluster headaches. Among the positive patients, about 65% are smokers and 6.5% have drinking behaviors. Regarding the relationship between sleep and CH, it is currently believed that sleep deprivation is one of the triggers of CH, and another theory is that the circadian rhythmicity of CH causes patients to passively change their sleeping habits, resulting in sleep deprivation (17, 18). Overall, poor lifestyle habits such as smoking and drinking may increase the frequency and severity of cluster headache attacks. Therefore, understanding these factors is important for the prevention and management of cluster headaches.

## Clinical manifestations and characteristics

Cluster headache is defined by periods of extremely acute one-sided headache or facial pain that can last anywhere from 15 min to 3 h. This is an important marker to distinguish CH from other headaches. It is regarded as one of the most severe types of pain. The intensity of pain surpasses that of labor, kidney stones, and shattered limbs (19). Cluster headache episodes exhibit both circadian and seasonal patterns. During the day, these episodes are most frequent at 2 a.m. In terms of the year, cluster headaches are most common in October, with a peak of occurrences occurring between April and October (20, 21).

Most cluster headache attacks are sudden and violent, often catching the patient off guard, but approximately 35% of patients are able to predict CH attacks, and it is worth noting that in these 35% of patients, the proportion of women, the prevalence and seasonal rhythmicity of pre-ictal symptoms, the frequency of cluster headache attacks per day, and the total number of attacks are higher (22). In a study of pre-episode symptoms of CH, a total of 327 patients participated, of whom PES occurred in 68 cases, and the most common symptoms were head and facial discomfort (23 cases, 33.8%), followed by anxiety and restlessness (15 cases, 22.1%), sleep problems (14 cases, 20.6%), fatigue/mild headache (non-ch) (11 cases, 16.2%), neck discomfort (9 cases, 13.2%), irritability/drowsiness (6 cases, 8.8%), acoustic phobia (4 cases, 5.9%), nasal congestion/osmophobia/muffled voices/photophobia/palpitations/palpitation (3 cases, 4.4%), seizure premonitions/dizziness/frustration/feeling of coldness/swelling of ears (2 cases, 2.9%), and shadow episodes (defined as minor pain and cluster episodes of short duration/toothache/hyperesthesia/diuresis/redeyes/food cravings/hyperactivity/constipation; 1 case, 1.5%) (23). This may help clinicians treat CH prophylactically and also better understand its pathophysiology. The headache is extremely severe. The nature of the pain is throbbing, drilling, or stabbing, which causes great distress to the patient.

Headache episodes can also have a severe impact on the patient’s mental state, with anxiety, an inability to meditate or concentrate, and the lifetime prevalence of depression in CH patients is 2.8 times higher than that of healthy individuals (24). This view was proved by another experiment (25). Because the pain cannot be relieved, the patient may engage in self-injurious behaviors, such as banging their head or hitting a wall, to relieve the pain (26). This not only seriously affects the patient’s daily life and work but also their mental health.

Another distinguishing feature of cluster headache is autonomic dysfunction (27). Pain is associated with the following ipsilateral cranial parasympathetic autonomic signs: conjunctival congestion; tearing; nasal congestion; rhinorrhoea; forehead and facial sweating; miosis; ptosis and/or eyelid edema; and/or restlessness or agitation (28). It is noteworthy that visual sensitivity is increased in patients with CH, especially during CH episodes; in two thirds of these cases, visual sensitivity was unilateral and predominantly ipsilateral to the cephalic side of the pain, which seems to help differentiate between cluster headaches and migraines, as photophobia in the latter is usually bilateral and more severe (29). In addition to affecting visual sensitivity, CH patients had significantly lower olfactory threshold scores and significantly impaired olfactory function compared to healthy controls (30). Patients with CH may experience these symptoms from autonomic nervous system disorganization due to cluster headaches. In addition, some patients may experience flushing, nausea, and vomiting. The onset of these symptoms is characterized by periodicity, with each attack lasting from a few minutes to several hours, followed by a relatively prolonged period of remission (31).

## Pathogenesis

Currently, research on the pathogenesis of CH has focused on the hypothalamus. A higher sympathetic tone was observed by stimulating the preganglionic fibers of the pterygopalatine nerve during episodes of CH with marked parasympathetic symptoms (32, 33). In addition, male patients with CH have reduced plasma testosterone levels and are often associated with reduced thyrotropin releasing hormone (34). A study comparing gender differences in patients with CH found that although the clinical phenotypes were similar, the daily seizure cycle was 1 h earlier in men with CH than in women, and there were more women than men with chronic CH (11). Furthermore, individuals with CH commonly exhibit reduced levels of melatonin during the night and disruption of their natural sleep–wake cycle (circadian rhythm) (35). Melatonin is acknowledged as a significant biomarker for controlling physiological rhythms, and the supraoptic nucleus of the optic chiasm in hypothalamic structures controls endogenous circadian rhythms (36). The supraoptic nucleus can control the generation and release of melatonin by establishing extensive connections with several hypothalamus nuclei, the pineal gland, and other structures (37). Thus, light sources may be the strongest trigger in the pathogenesis of CH through the retino-hypothalamic pathway.

Calcitonin gene-related peptide (CGRP) has been shown to be a migraine-inducing neuropeptide, and despite differences in headache phenotypes, migraine and cluster headache share some common features in certain places (e.g., response to triptans). Calcitonin gene-related peptides may exert their cluster headache-inducing ability in three different ways. First, this may occur through the vascular action of CGRP, which may involve neurogenic inflammation. Second, a CGRP receptor component has also been identified in the human trigeminal ganglion, which has been implicated as a possible site of action for CGRP receptor antagonists in migraine therapy. Third, pterygopalatine ganglion neurons express CGRP and its receptor components. Outflow from the pterygopalatine ganglion is thought to be the initiating mechanism for cluster headache attacks, and on-demand stimulation of the pterygopalatine ganglion is a new and effective treatment for cluster headache (38). Another study also confirmed that CH activity is associated with altered CGRP expression, with higher baseline levels of CGRP in patients with remission-phase CH compared to patients with chronic CH (39). Another study confirmed this idea and found that PACAP38 and vip-induced CH episodes were independent of plasma CGRP. Pituitary adenylate cyclase-activating peptide-38 (PACAP38) and vasoactive intestinal peptide (VIP) are both members of the vip glucagon, growth hormone-releasing factor-secretin superfamily, and their possible involvement in the initiation of attacks is supported by elevated plasma levels of PACAP and VIP during spontaneous CH attacks. CH pain was located in the trigeminal ophthalmic area, and concomitant intracranial autonomic symptoms suggested the involvement of trigeminal autonomic reflexes (TARs)-although whether the intracranial autonomic symptoms were caused by the pain or actually contributed to the pain is less clear. The trigeminal autonomic reflex consists of afferent trigeminal inputs and parasympathetic nerves that travel through the SPG via the supraspinal nucleus to the facial nerve, and it is possible that the trigeminal autonomic reflex interacts with the hypothalamus in the CH; however, the exact mechanism and sequence of events are not yet fully understood (40, 41).

Furthermore, the pathophysiology of CH may potentially entail inflammatory reactions in the cerebral pathways. The concept of chronic inflammation of the cavernous sinus has been proposed, suggesting that the cavernous sinus is the only anatomical site connecting the trigeminal and sympathetic blood supply and may play a role in the pathogenesis of CH through underlying cerebrovascular events (42). These findings offer fresh insights and guidance for a more profound comprehension of the development of CH.

## Whole process management

### Diagnostic procedures

#### Differential diagnosis

To properly recognize CH, it is important to have a more in-depth understanding of the autonomic nervous system symptoms in CH and to make the correct management when autonomic symptoms occur.

According to the third edition of the International Classification of Headache Disorders (ICHD-3), cluster headache is defined as recurrent unilateral severe headache in the orbital, supraorbital, or temporal regions lasting 15–180 min with cranial autonomic symptoms, irritability, or both. Cluster headache is classified into two subtypes: episodic, with episodic (paroxysmal) episodes lasting from 7 days to 1 year separated by periods of absence of at least 3 months’ duration in the past year; chronic, with episodes either without periods of relief or with periods of relief lasting less than 3 months in the past year; and chronic, with episodes either without periods of relief or with periods of relief lasting less than 3 months. Episodic, with attacks (paroxysms) lasting from 7 days to 1 year, separated by periods of absence (remission) lasting at least 3 months in the past year; chronic, with attacks that either have no remission or have been in remission for less than 3 months in the past year (43). Diagnosing cluster headache is a complex and delicate process, with a huge number of headache patients and a high rate of misdiagnosis, especially in the differential diagnosis between cluster headache and other headache disorders. When a patient with CH is seen in an outpatient clinic, the clinician can make a differential diagnosis by looking at the location and duration of the pain, as well as the presence or absence of autonomic symptoms. The pain in CH is usually unilateral and usually lasts for 15–180 min, which is a distinctive feature that differentiates it from other headache disorders. Migraine is usually a pain that lasts for more than 4 h. Paroxysmal hemicarania headache pain usually lasts 2–30 min. Sunct suna pain usually lasts 5–240 s or 2–600 s, and trigeminal neuralgia pain usually lasts a few seconds to 2 min (44, 45).

### Relevant examinations

#### Laboratory tests

There are no biomarkers that may be used to definitively diagnose CH, and laboratory testing is still in the development phase. The neuropeptide calcitonin gene-related peptide (CGRP) has attracted a lot of interest lately because of its extensive distribution in both neuronal and non-neuronal tissues of the body. CGRP plays a crucial role in the pathophysiology of migraine and cluster headache and blocking its function can alleviate neurogenic inflammation and sensitize pain pathways (46). Intravenous methylprednisolone and oral prednisone, used for short-term prophylaxis of episodic CH, led to a decrease in elevated plasma levels of CGRP in the blood of the external jugular vein, which was accompanied by a significant decrease in the frequency of CH onset, whereas patients with multiple sclerosis who served as a control group did not have alterations of these parameters, which seems to indicate that CGRP can be used as a biomarker for CH (47). Another study suggests that CH disease activity is associated with changes in CGRP expression (48). CGRP is not only a biomarker but also a therapeutic target for CH (49), and several studies have demonstrated the favorable efficacy of CGRP antibodies in the treatment of eCH, while they are ineffective in cCH. Galcanezumab is a highly specific and potent humanized immunoglobulin G monoclonal antibody targeting CGRP peptides. Galcanezumab is currently the only antibody approved for the treatment of CH, getting approved for the prevention of episodic and chronic migraine in the United States, Canada, the United Kingdom, and several European countries, and in June 2019, it has been approved for the prevention of eCH in the United States (50, 51).

There are also kynurenines, and the mechanisms of kynurenine involvement in CH have been described as follows: Glutamate has been shown to be involved in CH episodes. Glutamate glu acts on the n-methyl-d-aspartate (NMDA) receptor, which has an injurious sensitizing effect, and has been shown to be involved in the pathophysiology of CH. Kynurenine evolved from the catabolism of tryptophan (Trp). Some metabolites of the kynurenine pathway are neuroactive, and these metabolites are neurotransmitted by glutamatergic receptors, which modulate NMDA receptors. The kynurenines are also neuroactive. Trp catabolism, some metabolites of the kynurenine pathway are neuroactive, and these metabolites are neurotransmitted by glutamatergic neurotransmission, which plays an important role in the regulation of NMDA receptor function (52). In another study, human CH was found for the first time to be associated with abnormalities in the tryptophan metabolism kynurenine pathway (53, 54), which justifies the use of kynurenines as a direction for CH biomarker research.

#### Imaging examination

Currently, the main imaging research direction in CH is MRI, A study from China used resting-state functional magnetic resonance imaging (RS-fMRI) to assess the potential pathogenic involvement of low-frequency fluctuation fractional amplitude (faLFF) in CH. Specifically, faLFF is an indicator of the intensity of spontaneous localized brain activity. It is a signal that reflects the intensity of localized spontaneous activity in brain regions (55). The study showed that fALFF was reduced in the left cerebellum, left nucleus accumbens, left frontal lobe, left anterior cingulate and right postcentral gyrus in the left CH group, and in the right cerebellum, right cingulate, right superior parietal lobule, right inferior parietal lobule, right postcentral gyrus, and left precuneus in the right CH group (56), which further suggests that the complexity of the pathogenesis of CH may be due to the combined effects of multiple brain regions. Another study found that CH patients had weaker hypothalamic structural area covariance with frontal lobe (57). This observation also validates the findings of another study, who found that patients had less gray matter volume in the frontal lobes bilaterally than healthy controls and reported frontal hypometabolism in patients with CH (58, 59). Furthermore, the reduction in size of the left anterior superior temporal sulcus and the left lateral branch/glossal sulcus cortex in individuals with CH indicates a lack of proper adaptation of neuroplasticity in regions associated with social cognition. This may play a role in the development of psychiatric disorders and contribute to the severe disabling nature of CH (60). Aside from the frontal lobe, the cingulate gyrus, which has a vital function in regulating cortical responses to cluster headache, could be a potential target for neuromodulation in individuals who do not respond to medication treatment for cluster headache (61). Regarding the distinction between cluster headache and migraine by imaging, a study provides an answer: functional interactions between the left thalamus and parietal brain regions, including the precuneus gyrus and angular gyrus, are lower in patients with cluster headache compared to those with migraine (62).

In summary, the diagnostic process for cluster headaches requires a high degree of professionalism on the part of the physician. Physicians must utilize their extensive clinical expertise and practical experience to take a careful patient history and thoroughly examine the patient’s symptoms as well as laboratory data, with imaging helping to help them rule out secondary conditions. They must then perform a careful differential diagnosis to distinguish CH from other forms of headache. Only through this approach can doctors make an accurate diagnosis and subsequently develop a successful treatment plan for their patients.

### Therapeutic measures and management

Current treatments for CH focus on medications, oxygen therapy, and neurotherapy. We will describe each of these treatment options separately and conclude with recommended treatment management strategies.

#### Triptans

The main drugs currently used for the treatment of CH are the triptans, which official guidelines limit to twice daily use. Triptans can be administered in three ways: orally, subcutaneously, and as an intranasal spray. Some studies have shown that the mode of administration affects efficacy, with subcutaneous injections having the best therapeutic effect of the three modalities. Subcutaneous injection of sumatriptan resulted in complete pain relief within 20 min in 75% of subjects; sumatriptan nasal spray resulted in pain relief within 30 min in 47% of subjects (63). Prescribing tablets before nasal or injectable Triptans may delay optimal pain treatment. Oral administration is the least effective and the drug has a slow onset of action, so oral treatment is not recommended (64). Currently 6 mg subcutaneous sumatriptan or 20 mg sumatriptan and 5/10 mg intranasal zolmitriptan are recommended for the treatment of acute episodes of CH, and in his experiments, there was little difference in efficacy between 6 mg sumatriptan and 12 mg sumatriptan. Considering the possible side effects of tramadol (abnormal sensations, chest pains, sore throat, and sensation of fever), it is recommended to use 6 mg sumatriptan Tramadol is recommended to be used with 6 mg sumatriptan (65).

#### Oxygen therapy

Because of the vasoconstrictive properties of the triptans, they are contraindicated for use in patients with comorbid cardiovascular disease, making high-flow oxygen another treatment for acute CH (66). The exact mechanism by which oxygen therapy treats CH is uncertain. Possible mechanisms include inhibition of cranial parasympathetic pathways or trigeminal autonomic reflexes, modulation of neurotransmitters or neuropeptides such as calcitonin gene-related peptide to inhibit neurogenic plasma protein spillover, and cerebral arterial vasoconstriction (67). The currently recommended dose of oxygen therapy is inhalation of at least 12 L/min of 100% oxygen. In some cases, up to 15 L/min is required for 20 min using a non-rebreathing mask (68). Mo′s research proposes that the use of an oxygen concentrator absorbs surrounding air, filters out nitrogen, and produces an oxygen-enriched body that can be used as an alternative to using an oxygen tank as a source of oxygen when dealing with CH. The advantage of oxygen concentrators is that they do not need to be reoxygenated, but generally the machines have limitations in terms of maximum oxygen concentration (≤98%) and flow rate (≤5 L/min), and when higher oxygen concentrations and flow rates are required, connecting two oxygen concentrators can be an effective way of handling them (67). However, oxygen therapy does not provide pain relief for every CH patient; in a survey involving 3,251 CH patients, 13% were found to have complete relief after oxygen therapy, of which 41% were very effective, 27% moderately effective, 12% minimally effective, and 7% completely ineffective (69). This may have some correlation with the population, region or season.

#### Neuromodulation treatment

Neurotherapy is primarily used to treat refractory CH. Currently in neuromodulation for CH, it is divided into four main sections: vagus nerve stimulation (VNS), sphenopalatine ganglion stimulation, hypothalamic stimulation and occipital and cervical cord nerve stimulation. Vgus nerve stimulation has been approved by the FDA for the acute treatment of episodic cluster headache attacks and as an adjunctive treatment for the prevention of cluster headache (70). Most patients experienced pain relief within 15 min, with headache being the most common adverse effect (71), followed by dizziness, sore throat, and neck pain, and no serious adverse effects were observed (72). Sphenopalatine ganglion stimulation: it is an invasive treatment that requires implantation of a device in the pterygopalatine fossa below the maxilla, and studies have shown that it is more effective than VNS (approximately 67% of CH patients have pain relief within 15 min of treatment) (73), but it also has the disadvantage of being invasive, and there is a potential for postoperative adverse events, the most common of which are sensory disturbances, postoperative pain, and swelling (74). Furthermore, sphenopalatine ganglion stimulation demonstrated favorable results in treatment assessments lasting up to 24 months and shows promise as a therapeutic approach for chronic CH (75). Hypothalamic stimulation is a neurological treatment that is closely related to the underlying causes of CH. CH patients treated with this technique have a success rate of 88 per cent and have not encountered any significant negative effects (76). However, three serious adverse events, including subcutaneous infection, perioperative loss of consciousness with hemiparesis after trial simulation, and severe voiding syncope, occurred in another study on hypothalamic stimulation (77), which is a great test of clinician competence. In addition, occipital nerve stimulation is effective in reducing the frequency of CH episodes and the level of pain (78), a notion that is supported by a number of studies with concordant findings (79, 80), and there are fewer reports of adverse safety events with occipital nerve stimulation (81).

#### Anti-CGRP monoclonal antibody

The involvement of CGRP in the pathophysiology of headache disorders has been well established, making CGRP and its receptor a key target for the prevention of primary headache. Therefore, the emergence of multiple antibodies against CGRP and its receptors has provided new avenues of research for the pharmacologic treatment of CH (50). The emergence of galcanezumab has brought new hope for CH. As an anti-CGRP monoclonal antibody, galcanezumab reduces the symptoms of migraine by specifically binding to the CGRP receptor and blocking CGRP signaling. The unique mechanism of action of this drug, which has the advantages of being highly targeted and having few side effects, has attracted much attention from the medical community (82). According to a study published in a prestigious medical journal, galcanezumab has shown remarkable results in the treatment of migraine headaches. One study shows that the majority of patients treated with galcanezumab experienced significant headache relief in as little as 3 weeks (83, 84). This was confirmed in another study in which the median time to remission after the first 240 mg galcanezumab treatment was 17 days and the proportion of patients with a 50 per cent reduction in the number of headache attacks per week from baseline at week 3 was 78.8 per cent; 91.5 per cent of patients with acute headache received only one GT treatment, and 74.5 per cent of patients with ECH experienced remission 1 month after GT treatment; the fewest adverse events occurred in patients using the 240 mg dose of galcanezumab. The lowest number of adverse events occurred in patients treated with the 240 mg dose of galcanezumab (85). However, currently available studies indicate that galcanezumab is only effective in the prevention of eCH and has not been found to be effective in the prevention of cCH (86). Another CGRP antibody is Fremanezumab, a human IgG2 monoclonal antibody, and two trials on fremanezumab have failed in trials that have been conducted on fremanezumab for the prevention of episodic and chronic cluster headaches. Each trial demonstrated an inability to reduce the number of CH attacks per week (87).

Overall, the emerging therapeutic directions are still in the research stage, and their drawbacks are obvious, i.e., the lack of clinical data, but recent studies have provided clinicians with innovative ideas for choosing treatments for CH, especially for refractory CH, for which studies are now available that show favorable therapeutic outcomes.

## Conclusion

Cluster headache is a common and severe primary headache disorder. The pathogenesis of cluster headache is still in the research phase, but many studies have pointed the way to the study of cluster headache pathogenesis. The identification of biomarkers and imaging assays has further advanced these studies, improving our ability to diagnose, prognose, and monitor treatment response in real time. The inherent heterogeneity of neurologic disorders, the selective permeability of the blood–brain barrier, and ethical considerations in clinical trials are formidable hurdles to overcome. The strategies we give for managing the full course of the disease still need to be individualized and studied. In conclusion, many new avenues for the treatment of CH are now available, and we can further advance these studies to improve the quality of life of CH patients.

## Author contributions

X-HX: Conceptualization, Writing – original draft, Funding acquisition, Investigation, Resources, Supervision. Y-ML: Visualization, Writing – original draft, Data curation, Investigation, Methodology. L-NR: Data curation, Formal analysis, Investigation, Project administration, Writing – original draft. X-FX: Investigation, Methodology, Supervision, Validation, Writing – original draft. Y-LD: Investigation, Methodology, Project administration, Software, Writing – original draft. C-QJ: Conceptualization, Project administration, Software, Supervision, Visualization, Writing – original draft, Writing – review & editing. R-RY: Conceptualization, Data curation, Methodology, Software, Visualization, Writing – original draft, Writing – review & editing.
